# DPM dispersion inside a single straight entry using dynamic mesh model

**DOI:** 10.1007/s40789-017-0179-9

**Published:** 2017-08-03

**Authors:** Yi Zheng, Ying Li, Magesh Thiruvengadam, Hai Lan, Jerry C. Tien

**Affiliations:** 1 Missouri University of Science and Technology, Rolla, MO USA; 2 Clean Air Power Inc., Poway, CA 92064 USA; 3 Monash University Clayton Campus, Wellington Road Clayton, VIC 3800 Australia

**Keywords:** CFD, DPM, Dynamic meshing, Piston effect, Backflow, Buoyancy effect, Layering of DPM

## Abstract

Three-dimensional simulations of diesel particulate matter (DPM) distribution inside a single straight entry for the Load-Haul-Dump loader (LHD)-truck loading and truck hauling operations were conducted by using ANSYS FLUENT computational fluid dynamics software. The loading operation was performed for a fixed period of 3 min. The dynamic mesh technique in FLUENT was used to study the impact of truck motion on DPM distribution. The resultant DPM distributions are presented for the cases when the truck were driving upstream and downstream of the loading face. Interesting phenomena were revealed in the study including the piston effect, layering of DPM in the roof region, and backflow of diesel exhaust against ventilation. The results from the simulation can be used to determine if the areas inside the face area and straight entry exceed the current U.S. regulatory requirement for DPM concentration (>160 µg/m^3^). This research can guide the selection of DPM reduction strategies and improve the working practices for the underground miners.

## Introduction

For underground mines, self-propelled diesel equipment is preferred due to its high fuel efficiency, ease of maintenance, reliability and durability. However, emission from the tailpipe and its subsequent distribution in the underground mine are of growing concerns for miners.

Diesel particulate matter (DPM) is the particulate by-product of diesel exhaust and it can exist in different modes with different size distributions (5 nm–10 µm). Of these particles and chain aggregates, more than 90% are sub-micrometre in size, normally less than 1 micrometre in diameter. Due to the small size and adsorb ability [more than 1800 different organic compounds and potentially toxic hydrocarbons were identified (CFR 2001)], it can be breathed into the alveolar region of the lungs of miners and cause acute and chronic health problems such as asthma, eye and nose irritation, headaches and nausea (Kahn and Orris 1988; Wade and Newman 1993; Rundell et al. 1996) to long term carcinogenic effects (NIOSH 1988; EPA 2002).

For underground coal mines, diesel engines used underground are divided into three categories under Mine Safety and Health Administration (MSHA) regulations: “permissible”, “nonpermissible heavy-duty equipment, generators, and compressors” and “nonpermissible light-duty equipment”. Equipment under each category is required to emit no more than a certain amount of DPM per hour (30 CFR 72.D 2014); otherwise, it will not be allowed to operate underground.

For underground metal/non-metal mines (M/NM), MSHA regulations limit a miner’s personal exposure to DPM no more than 160 µg/m^3^ of total carbon (TC) for an average 8-h equivalent full shift (effective from May 20, 2008) (30 CFR 57.5060 2014). Today, there are still mines that cannot meet this regulation limit.

To control DPM hazards, two types of engineering controls have been commonly used. One is curtailment of emissions at the source, which includes proper diesel engine selection and maintenance (Anyon 2008; McGinn et al. 2010), use of alternative fuels (Zannis et al. 2009; Bugarski et al. 2010), and exhaust gas treatment devices (Shah et al. 2007; Bugarski et al. 2009), e.g., diesel particulate filters (DPF). The other is reducing exposures after release of diesel emissions into the work environment—dilution by the mine ventilation system, an enclosed equipment cab with filtered breathing air (environmental cab) and personal protective equipment. Administrative controls can be used to complement both engineering approaches: e.g., a no-idle policy can be used to reduce emissions, limiting number of the vehicles in ventilation split can be used to reduce exposures (Cecala et al. 2005; Noll et al. 2008; MSHA 2013).

Experience (Bugarski et al. 2011) showed that no single strategy can solve all DPM problems and a combination of several measures needs to be implemented in the field to attain compliance. To achieve an effective, efficient, and economical control scheme, an understanding of DPM behaviour in the mining environment can be very useful in selecting the control strategies and training the miners. Numerical simulations using computational fluid dynamics (CFD) can be used for that purpose to reveal DPM distribution based on laboratory experiments and field studies.

CFD simulations have been successfully used in environment control research to detect spontaneous combustion and apply inertisation in gob areas (Ren et al. 2005; Yuan and Smith 2007), study airflow patterns and gas concentrations in continuous miner operations or heading development (Hargreaves and Lowndes 2007; Wala et al. 2007; Kollipara et al. 2012; Zhou et al. 2015), investigate scrubber intake designs for longwall dust control (Ren and Balusu 2008), and estimate a mine’s damage status by tracer gas and simulation after a disaster (Xu et al. 2013). In the study of occupational respiratory protection, Lei et al. (2013) applied the CFD simulation approach for the prediction of leakage between an N95 filtering facepiece respirator and a headform. The thermal impacts on the human model from the respirator was also simulated with CFD.

Simulation of different gases from diesel engines, like CO_2_, CO, O_2_, and NO, was conducted to evaluate the locations of tailpipe and auxiliary ventilation system to improve the working environment (Kurnia et al. 2014). Simulation directly addressing DPM dispersion in underground mines was carried out by Zheng and Tien (2008), in which DPM was considered to behave like a gas. Subsequent studies showed that it gave good quantitative agreement with practical accuracy for the DPM distribution and successfully identified the DPM affected areas above the threshold limit (Zheng et al. 2011). Afterwards, Zheng et al. (2015a) evaluated four different push–pull ventilation systems to improve a deep dead end entry working environment and studied DPM distribution based on an industrial field study (McGinn et al. 2004; Zheng et al. 2015b). In the present study, DPM emission was also treated as a gas to examine its dispersion inside an underground single straight entry.

Since some vehicles, like trucks and LHD, move around underground most of the time, it is essential that the motion and the effect of the motion on mine ventilation and DMP distribution to be investigated. The motion effect was investigated previously in a deep dead-end entry (Thiruvengadam et al. 2016), where loading operation was conducted with a push-pull local ventilation with and without DPF. That scenario of 90 m deep dead-end entry perpendicular to the main entry may not be an often encountered working face for the miners and mining engineers. Therefore, the results of the study may have limit to provide guidance in the real-world. In this study, the motions effect in a straight main entry face area with a LHD-truck loading and a truck hauling operation afterwards were examined. It is a more regular working environment than the deep dead-end entry. And the results are expected to be more applicable to the mining industry with similar working strategies.

The study consisted of two scenarios (1) the truck driving upstream and (2) the truck driving downstream after the LHD-truck loading operation. This study clearly revealed a combination of piston effect, layering and backflow of the high DPM concentration plume. This study can help understand the effect of vehicles’ motion on DPM distribution and choose the best working practice for the miners.

## Problem statement and CFD modelling

### Statement of the problem

**Fig. 1:**
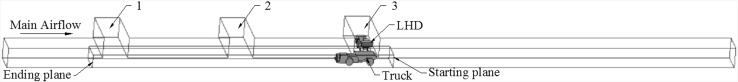
Starting location of scenario 1

**Fig. 2:**
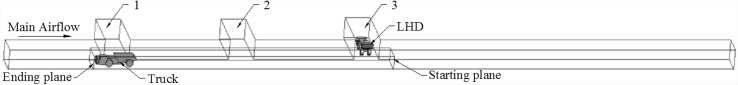
Ending location of scenario 1

**Fig. 3:**
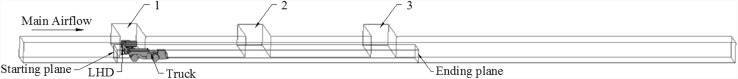
Starting location of scenario 2

**Fig. 4:**
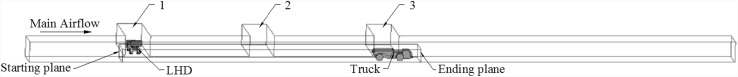
Ending location of scenario 2

The schematic of the straight entry face areas was designed based on a typical entry in an M/NM operation in the US. It contained three face areas and the loading operation was taking place in either face 1 or face 3 (Figs. 1, 2, 3, 4). The LHD was assumed to be loading for 3 min, and then the ore was hauled away by the truck. The truck was moving at a constant speed of 1 m/s either upstream against the main airflow (scenario 1) or downstream with the same direction as the main airflow (scenario 2). The haul distance of the truck was about 60 m within a region between starting plane and ending plane as shown in the figures. During the truck motion, the LHD was assumed to be idling.

In the mining scenarios here, the LHD was operated in a small face area to load the truck instead of driving a long distance to dump into an ore pass. Therefore, to simplify the problem, the LHD was considered as a stationary diesel engine. Only the motion of the truck was studied.

The straight entry inlet on the left provided 19.5 m^3^/s of fresh air flowing from left to right into the computational domain. The straight entry measured 6 m × 5 m × 210 m, width × height × length, while the three faces had the dimension of 6 m × 5 m × 10 m, width × height × depth. The distance between the adjacent faces was 24 m and the straight entry upstream of face 1 measured 21 m.

### CFD modelling

To study the DPM phenomenon, the ANSYS FLUENT CFD program was used. The physical properties of fresh airflow treated as constants are listed as follows: inlet temperature, *T*_0_ = 27 °C; specific heat (*C*_p_), 1006 J/kg ^o^C; dynamic viscosity (*μ*), 1.789 × 10^−5^ kg/m s, and thermal conductivity (*k*), 0.0242 W/m ^o^C. In the simulation, the air was considered as dry air, which the humidity variations in the model was not accounted.

The density variation in the fluid due to temperature gradient between the air and diesel exhaust was calculated using the incompressible ideal gas model within ANSYS FLUENT. In the presence of gravity, this density gradient resulted in buoyancy flow. The DPM concentration inside the single dead end entry was determined using the species transport model within FLUENT where diesel particulates were treated as gas (continuous phase) and the material used as a surrogate for DPM was n-octane vapour (C_8_H_18_) with density (*ρ* = 4.84 kg/m^3^), specific heat (*C*_p_ = 2467 J/kg ^o^C), thermal conductivity (*k* = 0.0178 W/m ^o^C) and dynamic viscosity (*μ* = 6.75 × 10^−5^ kg/m s). The species transport model allowed the two species, air and DPM, to diffuse and form a mixture. The mixture properties were derived using the incompressible ideal gas law for density, the mixing law for specific heat, thermal conductivity and viscosity. The mass diffusivity between air and DPM was assumed to be a constant with *D* = 5e^−6^ m^2^/s. The chemical reaction between the species was not considered in this study. The settings were derived from previous experiment validation study mentioned above (Zheng et al. 2011, 2015b).

The airflow inside the computational domain was solved in an Eulerian frame as continuous phase using the time averaged three-dimensional turbulent transient Navier–Stokes, energy, and continuity equations using the finite volume method. An additional non-reacting two species transport equation (DPM and Air) was solved in an Eulerian reference frame (since both air and DPM were treated as continuous phase) to determine the mass fraction of DPM. The turbulence in the flow was modelled using the standard *k* − *ε* turbulence model with standard wall functions for near wall treatment. Boundary conditions used to determine the DPM distribution inside the single straight entry are listed in Table 1. The emission rates for truck and LHD were calculated from a Diesel Emission Evaluation Program (DEEP) field study (McGinn et al. 2004) and were reported in the DPM CFD research carried out by Zheng et al. (2015b). For the LHD, the tailpipe is located at the right rear of the vehicle and pointing backwards; for the truck, the discharge port is under the vehicle and placed at the right front of the truck, pointing towards the floor.

**Table 1:** Boundary conditions used for DPM simulation

Boundaries	Boundary conditions
Main airflow-inlet	Velocity (normal to boundary) = 0.65 m/s, *T* = 300 K, DPM mass fraction = 0.0
Main airflow-outlet	Outflow or fully developed boundary conditions
LHD-tailpipe	Velocity (normal to boundary) = 24.1 m/s, *T* = 594 K, DPM mass fraction: 7 × 10^−6^
Truck-tailpipe	Velocity (normal to boundary) = 27.5 m/s, *T* = 644 K, DPM mass fraction: 7 × 10^−6^
Walls	No slip boundary conditions Adiabatic walls (heat flux = 0) Zero diffusive flux

### Mesh generation and solution methodology

**Fig. 5:**
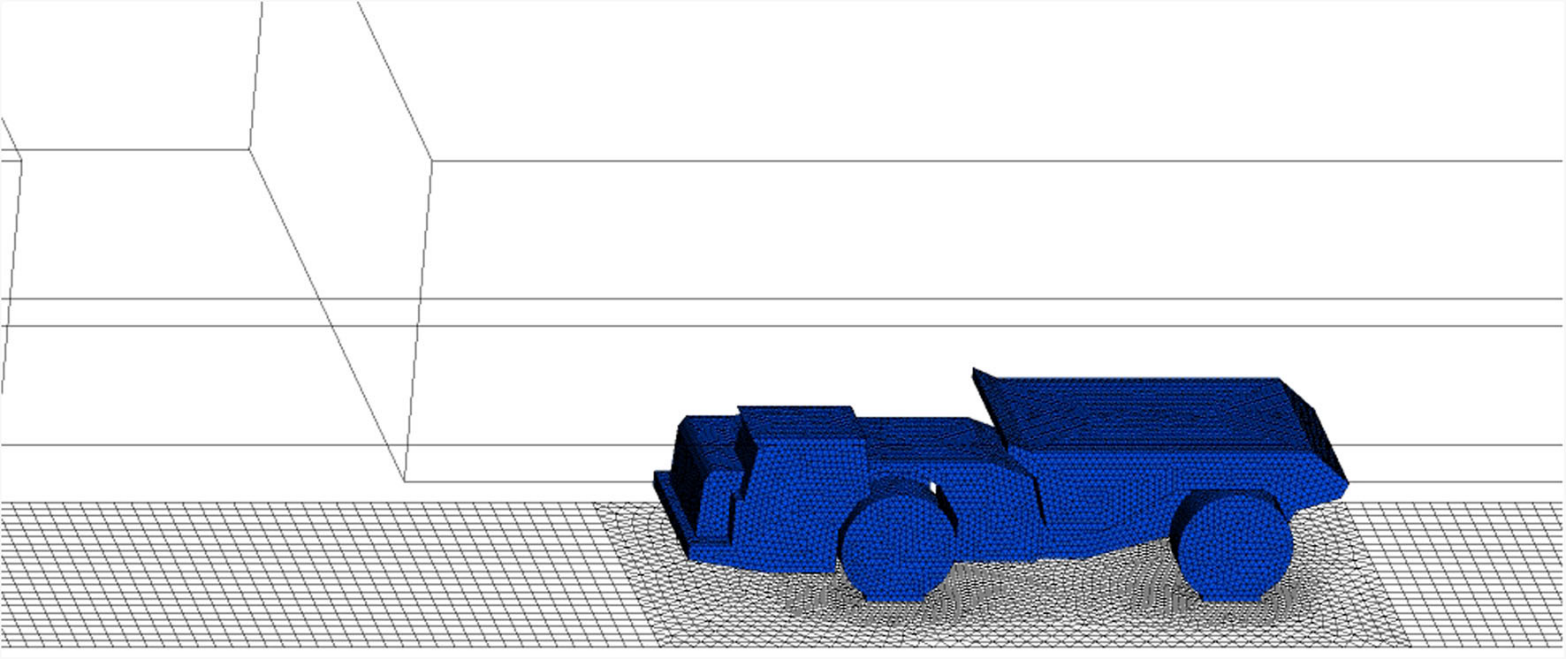
Mesh generation for vehicle’s motion study

In this study, the truck was assumed to move with a constant speed of 1 m/s. To account for the vehicle motion, a technology called “dynamic mesh simulation” is available in FLUENT which permits the motion of some components in a domain, while the other components remain stationary. In this study, the motion of the truck was prescribed by specifying the linear speed and the direction of motion using the UDF (User Defined Function) option in FLUENT. The mesh for the computational domain was obtained using FLUENT’s pre-processor GAMBIT as shown in Fig. 5. A total of 750000 cells (both hexahedral and tetrahedral) were generated. The mesh generation was made by ensuring a high density near the LHD/truck and in the bounding wall regions where high gradients exist in order to ensure simulation accuracy. During mesh generation the equi-size skew was monitored and maintained at a value less than 0.8.

FLUENT expects the description of the motion to be specified on different moving and non-moving cell zones. Furthermore, regions that are deforming due to motion on their adjacent regions must also be grouped into separate zones. As shown in Fig. 5, the truck was defined as a moving truck zone and meshed with tetrahedral cells. The neighbouring zones were meshed with hexahedral cells to facilitate the new mesh generation. Whenever the moving truck zone moved to a new location, one layer ahead of the zone collapsed while one layer behind it added. This method is called a dynamic layering approach in FLUENT.

Detailed descriptions of the CFD code and the solution procedures can be found in the FLUENT 12.0 documentation (ANSYS 2014). The unsteady flow was calculated using a time step Δ*t* = 0.1 s for both loading and hauling operations for the total time duration of 240 s (180 s for loading operation plus 60 s for hauling operation). The convergence criterion required that the scaled residuals be smaller than 10^−4^ for the mass, momentum, turbulent, and species transport equations and smaller than 10^−9^ for the energy equation. Calculations were performed on the Numerical Intensive Computing (NIC)-Cluster using 16 processors and the CPU time for converged solution was approximately 24 h to obtain the results for both loading and hauling operations.

## Results and discussion

**Fig. 6:**
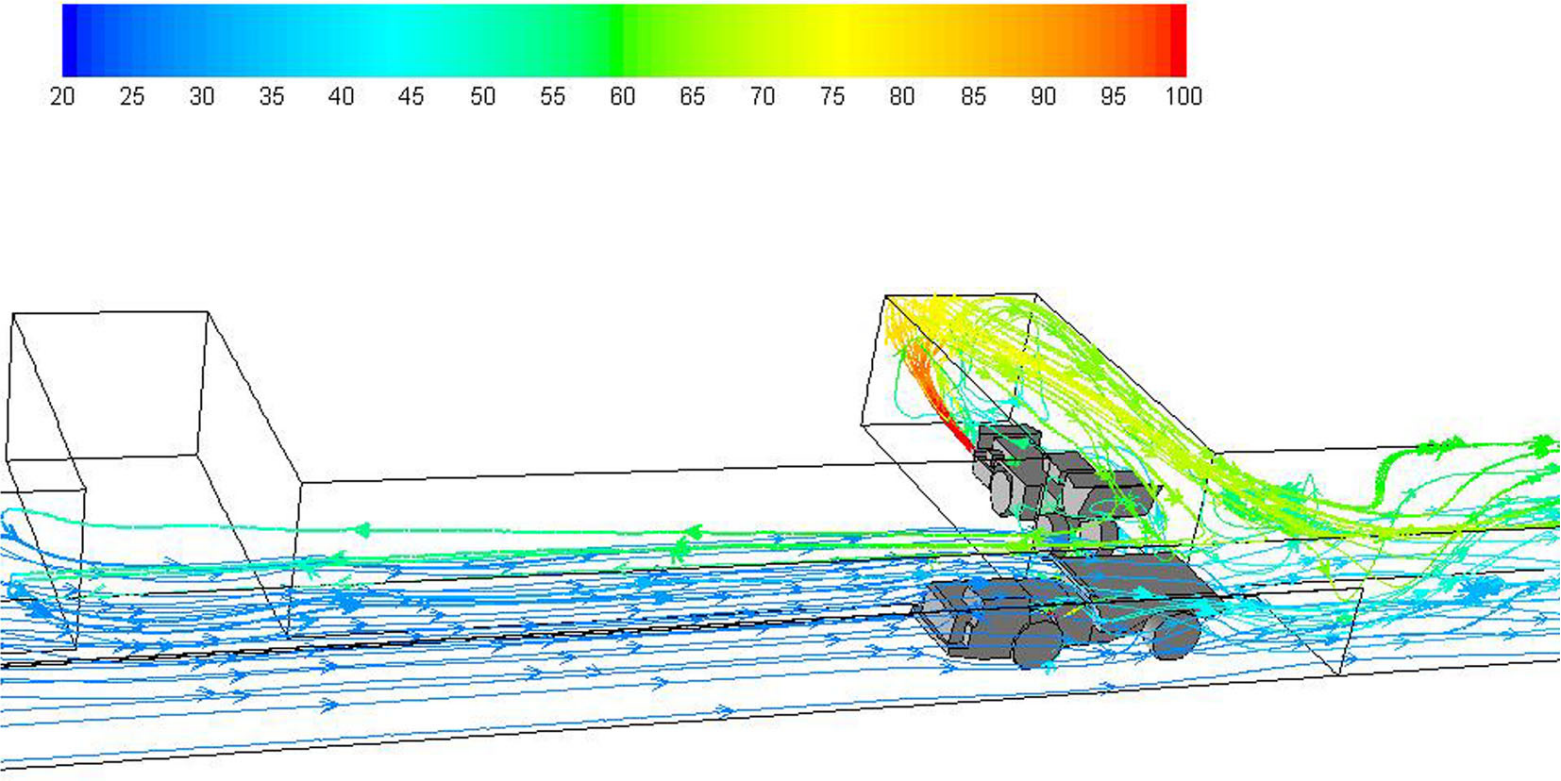
Pathlines colored by temperature (°C) showing general flow features during the loading operation (legend 20–100 °C)

**Fig. 7:**
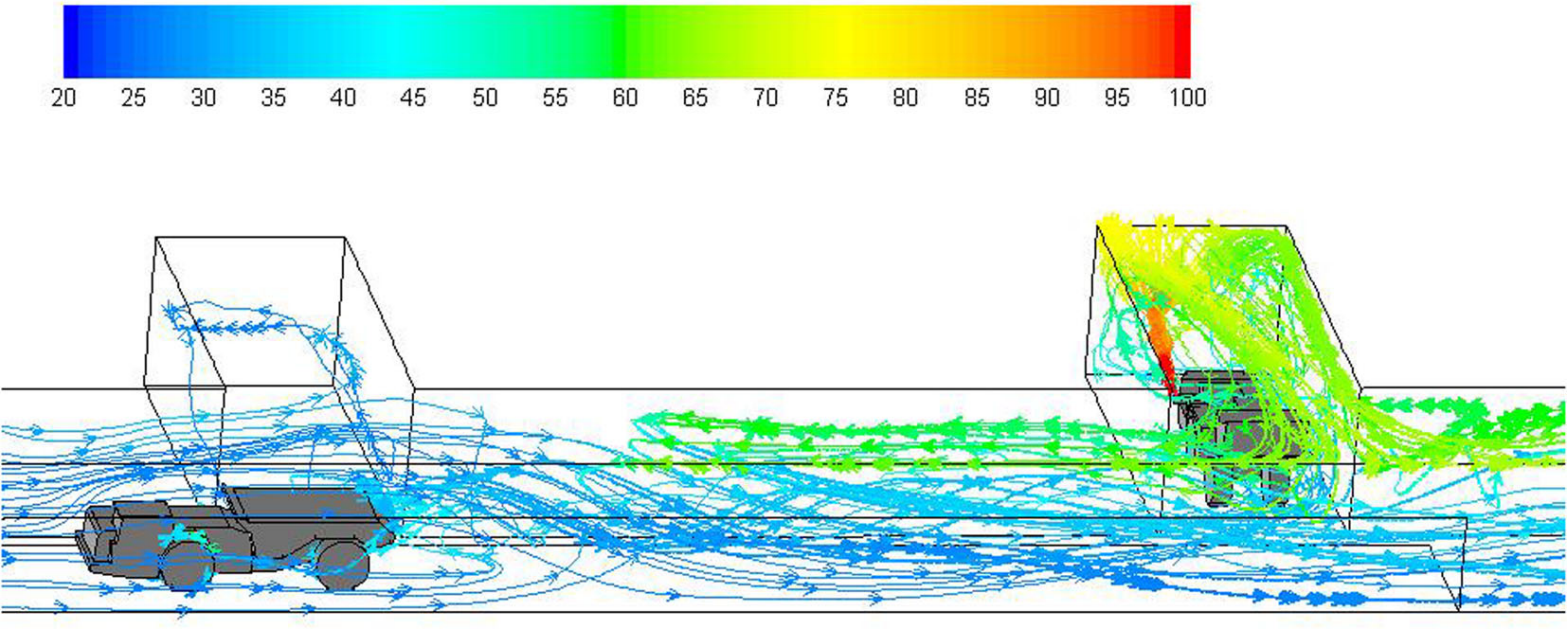
Pathlines colored by temperature (°C) showing general flow features during the hauling operation (legend 20–100 °C)

Numerical simulation of stationary loading and the subsequent dynamic hauling operation was carried out for the computational domain shown in Figs. 1, 2, 3 and 4. For both scenarios 1 and 2, the main airflow flowed from left to right. The flow features for diesel engines in this straight entry are shown in Fig. 6 during loading operation and in Fig. 7 during hauling operation. It can be observed that the main airflow is flowing from left to right by the pathlines, indicating the general ventilation status in this straight entry.

However, two more important flow features are revealed in Figs. 6 and 7: one is the layering of the exhaust flow in the roof region inside the face and connection region of the face with main entry; the other is the backflow of diesel exhaust against main ventilation. Both features are caused by the low velocity of the main airflow and the buoyancy effect.

In Figs. 6 and 7, it can be observed that hot diesel exhaust was injected out horizontally from the LHD tailpipe at high temperature. After that, the exhaust flow hit the face and then bounced back toward the straight entry. During the process, the hot and lower density exhaust flow moved toward the roof region as shown in the figures due to the buoyancy effect in the presence of gravity.

The exhaust flow occupied most of the roof region inside the face and gradually spread out at the intersection where the face entry met the straight main entry. At this place, the still hot exhaust clung to the roof and wedged the main airflow out of the ceiling region. There was heat exchange between the exhaust flow and the cold ventilation flow, during which the exhaust lost heat and became heavier and then mixed with the surrounding main airflow; at the same time, the ventilation flow close to the exhaust gained the heat and moved upward and blocked the further dilution of the exhaust gas from the main ventilation. Overall, the low speed of the main airflow was not strong enough to sweep the exhaust at the intersection.

As the diesel exhaust continued to be ejected from the engine tailpipe, it continued to spread out in all directions at the intersection due to the weak main ventilation flow. At the end of the loading operation as shown in Fig. 6, the exhaust flow can even backflow to the upstream of face 2 as indicated by the pathlines.

Figure 7 shows the general flow pattern (pathlines) developed during the hauling operation with the truck about halfway through the dynamic mesh zone. The flow pattern was similar to the loading operation in the face area, except that as the vehicle moved upstream at a speed of 1 m/s, there was more turbulence and intensive recirculation at the back of the truck due to the piston effect. This more intensive turbulent flow reversed the backflow of diesel exhaust and made the backflow effect reduce in the upstream regions.

Similar flow pattern were observed in scenarios 1 and 2. Therefore, only the truck hauling against the main airflow are shown below.

### Scenario 1: loading operation

For the loading scenario 1, the truck was facing the fresh airflow during loading and driving against (into) the fresh airflow after loading.

**Fig. 8:**
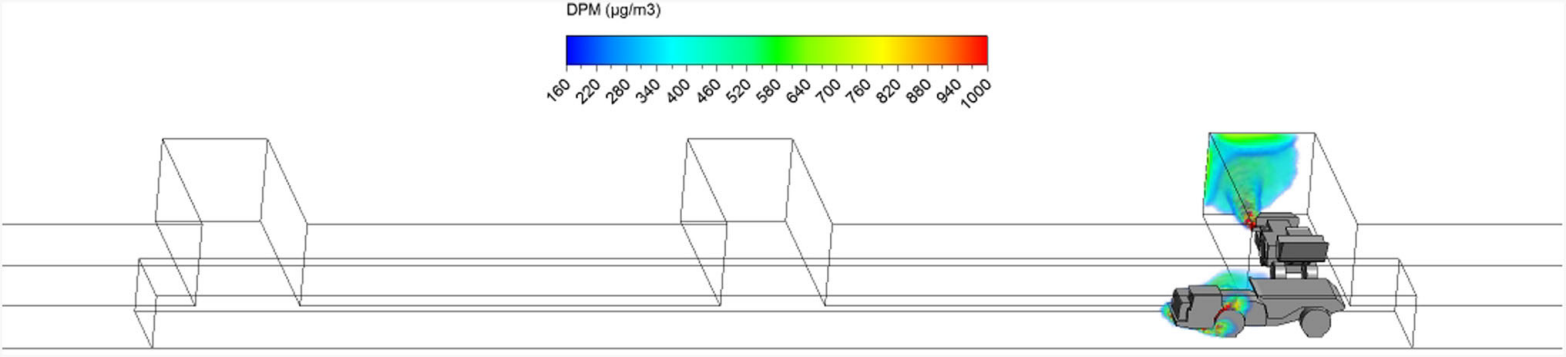
DPM distributions in scenario 1 during the loading operation at 3 s

**Fig. 9:**
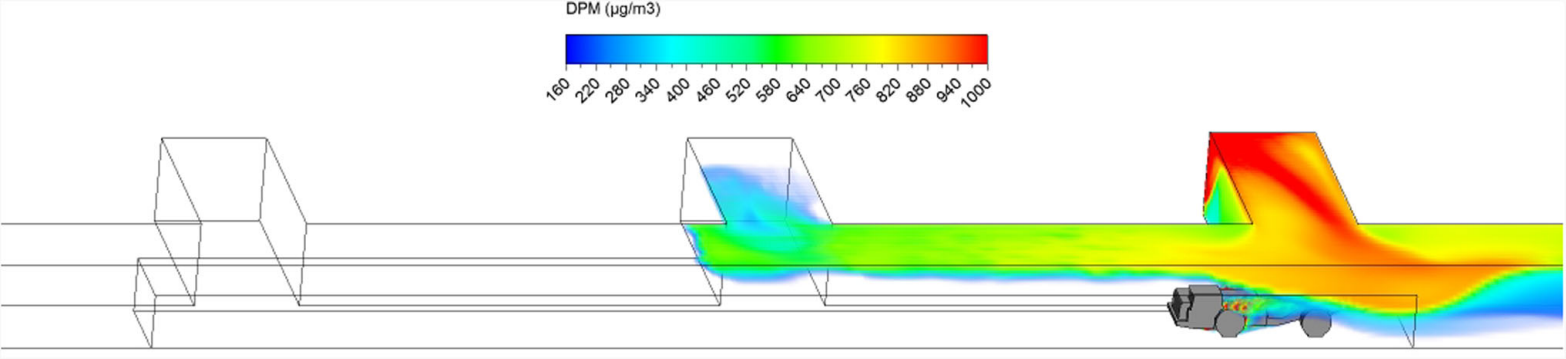
DPM distributions in scenario 1 during the loading operation at 180 s

The loading operation took place for exactly 3 min. The DPM distribution (≥160 µg/m^3^) in the straight entry and face region at the beginning (3 s) and at the end of the loading operation (3 min) are shown in Figs. 8 and 9. It can be observed that the high DPM plume spread from the tailpipes of the LHD and truck, gradually occupied the whole face region and the roof area in the straight entry. The backflow of DPM mentioned earlier can be more clearly seen in Fig. 9, showing it flowed to the roof level at the intersection and face area upstream of face 2.

At the end of the loading operation (180 s), it was observed that the loading area and the immediate downstream regions were filled with high DPM levels (Fig. 9). The LHD operator and the miners working in the vicinity need additional protection to lower their DPM levels. Even the miners upstream of the loading face, if working at roof level like the scaler to secure the roof, should be aware of the possible DPM pollutants and wear personal protective equipment. However, the truck driver did not seem to be affected by the high DPM plume.

### Scenario 1: hauling operation

**Fig. 10:**
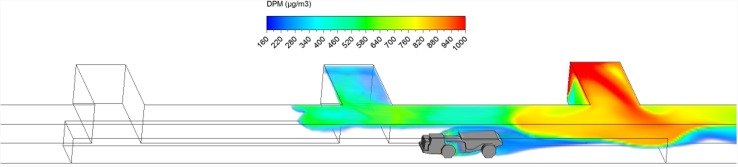
DPM distributions in scenario 1 during the hauling operation at 195 s

**Fig. 11:**
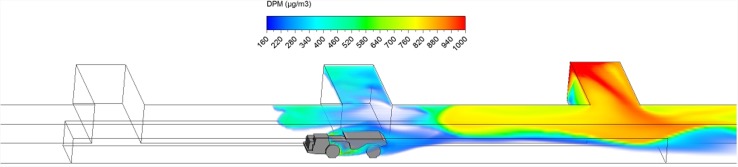
DPM distributions in scenario 1 during the hauling operation at 210 s

**Fig. 12:**
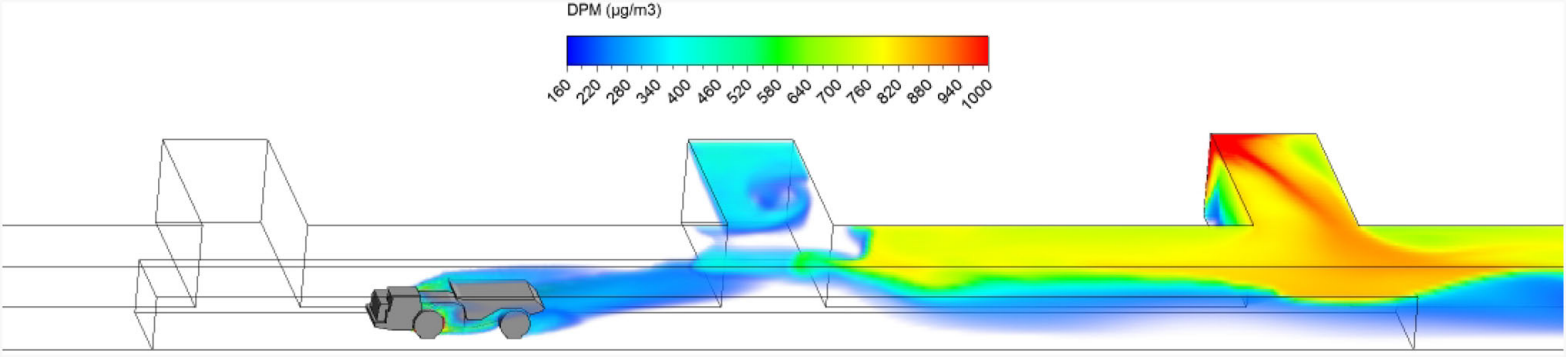
DPM distributions in scenario 1 during the hauling operation at 225 s

**Fig. 13:**
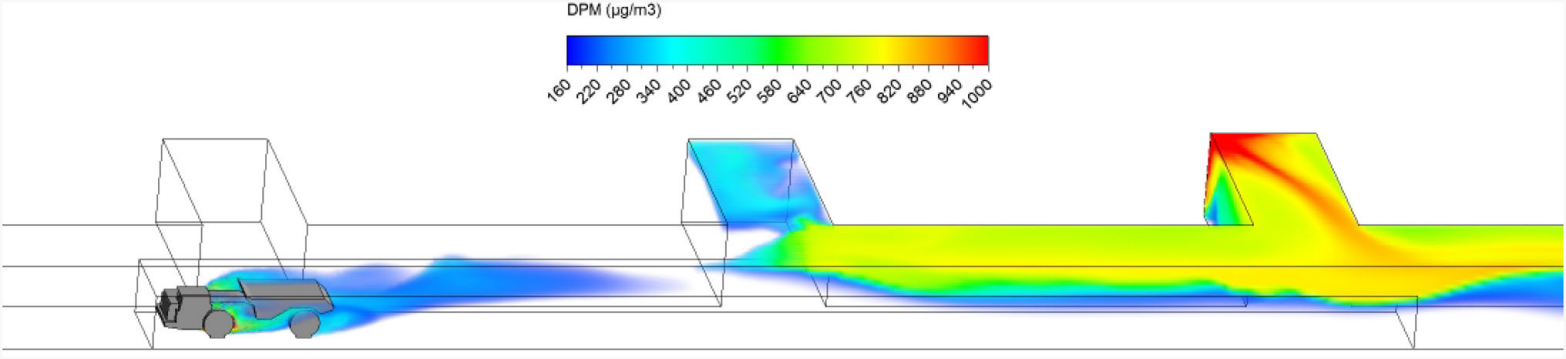
DPM distributions in scenario 1 during the hauling operation at 240 s

Hauling started as soon as ore loading was completed. After loading, the truck drove at a uniform speed of 1 m/s against the fresh airflow in the straight entry discharging DPM at a constant rate along the way. As shown in a series of time-lapsed simulated results showing truck in four different locations in Figs. 10, 11, 12 and 13, it can be observed that the DPM plume produced a “tail” about 25 m long downstream from the truck engine and curved toward the roof due to the buoyancy effect, but the truck driver was still outside the high DPM plume. At the same time, the LHD was still operating inside the face area where the DPM levels were very high. Other DPM controls would be needed to improve the LHD operator’s working conditions.

As the haulage truck (2.7 m × 2.5 m × 10 m, width × height × length) started driving upstream, it blocked part of the straight entry airway and increased the airflow speed elsewhere in the same cross-sectional plane. As a result, the high DPM plume that hung on the roof upstream of the loading point was diluted by the higher ventilation speed as the truck drove by as shown in Figs. 10 and 11.

The motion of the truck caused a piston effect so that behind the truck, as air was pushed away, a vacuum was created that sucked the high DPM plume into that region as the plume flowed up due to the buoyancy effect. The piston effect here made the DPM plume flattened as compared with previous study where the truck was assumed stationary (Zheng et al. 2015b). The overall effect of ventilation, buoyancy/gravity force, and piston effect can cause different shapes of the high DPM plume. Incorporating the vehicle’s motion can definitely increase the accuracy of the prediction and help to design and prepare the working practices.

### Scenario 2: loading operation

For loading scenario 2, the truck was facing downstream and then was driven into the exhaust flow after loading.

**Fig. 14:**
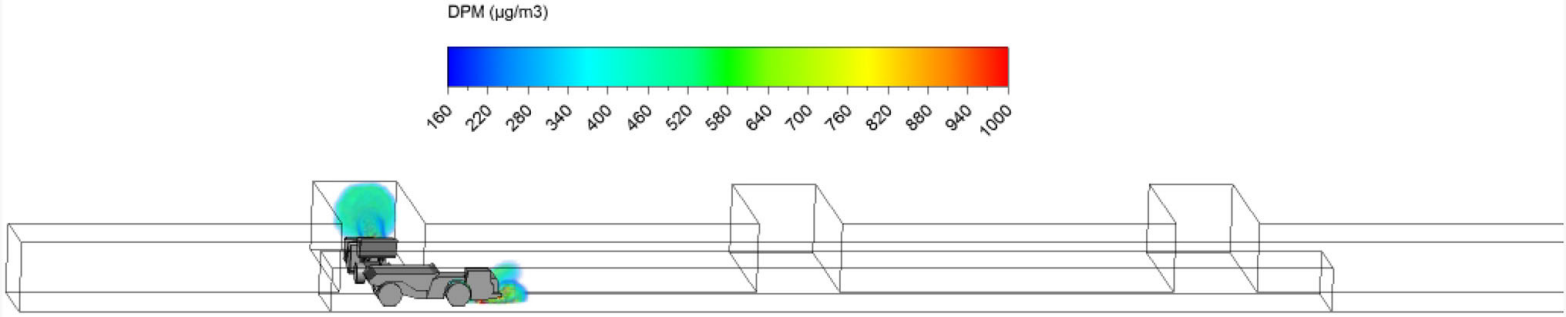
DPM distributions in scenario 2 during the loading operation at 3 s

**Fig. 15:**
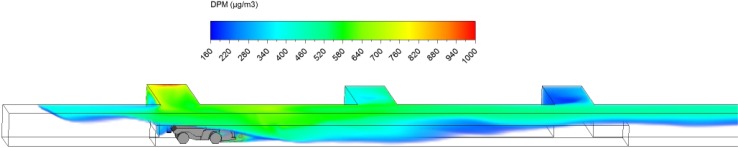
DPM distributions in scenario 2 during the loading operation at 180 s

The DPM distribution (≥160 µg/m^3^) in the straight entry and face region at the beginning (3 s) and at the end of the loading operation (3 min) are shown in Figs. 14 and 15. Similar to scenario 1 in the loading operation, it can be observed that the high DPM plume spread from the tailpipes of the LHD and truck, gradually occupying the whole face region and the roof area in the straight entry. The high DPM exhaust flow can also backflow upstream as can be clearly seen from Fig. 15, indicating the weak main airflow was not effective in sweeping the produced DPM downstream.

At the end of the loading operation (180 s), a high DPM concentration plume occupied the whole face area and the coloured regions upstream and downstream of the face (Fig. 15). The LHD operator, the close-by miners and roof level working miners upstream need additional protection as described in scenario 1. In scenario 2, even the truck driver was affected by the high DPM plume since part of the cab was located in the high DPM plume. An environmental cab or personal protective equipment is recommended to provide a healthy working condition for the truck driver.

### Scenario 2: hauling operation

**Fig. 16:**
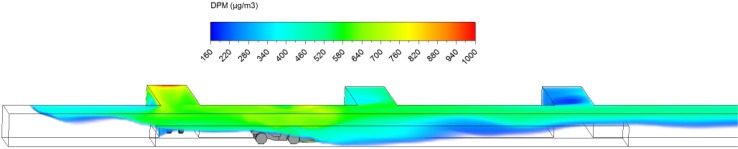
DPM distributions in scenario 2 during the hauling operation at 195 s

**Fig. 17:**
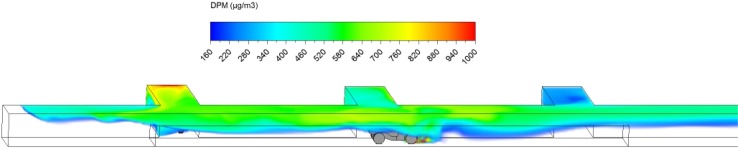
DPM distributions in scenario 2 during the hauling operation at 210 s

**Fig. 18:**
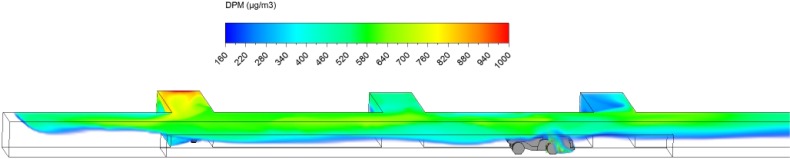
DPM distributions in scenario 2 during the hauling operation at 225 s

**Fig. 19:**
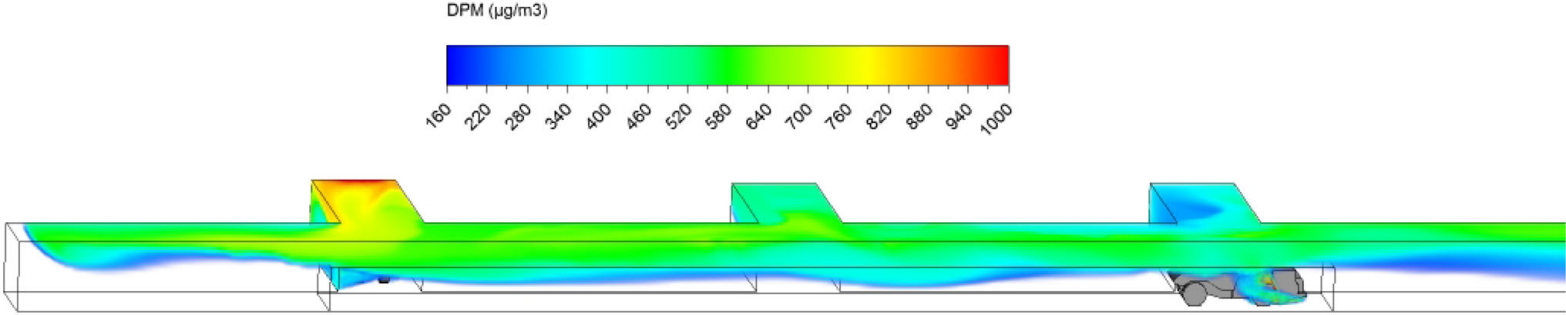
DPM distributions in scenario 2 during the hauling operation at 240 s

Similar to the scenario 1 hauling operation, it can be observed from Figs. 16, 17, 18 and 19 that the LHD operator was always working in high DPM face area and need additional protection, like the environmental cab or personal protective equipment, all the time.

For the truck, instead of driving out of the high DPM plume as in scenario 1, it drove downstream into the high DPM fumes produced during loading. Since the truck drove at 1 m/s, a little higher than the main airflow speed (0.65 m/s), it chased and pushed the high DPM plume in front of the vehicle. The piston effect seemed to push away the DPM fume close to the ground level. But during the process, the truck driver were affected by DPM plume produced by the upstream LHD engine and its own engine. A sealed environmental cab or personal protective equipment is suggested for the truck driver in scenario 2.

## Conclusions

Numerical simulation of DPM distribution inside a straight entry was carried out for a LHD-truck loading and truck hauling operation. The simulation included cases of the diesel truck hauling upstream and downstream of the loading point with a constant driving speed.

This study revealed the combined effects of vehicle motion, buoyancy/gravity force, and low ventilation speed. DPM dispersion was affected and interesting phenomena were revealed: a piston effect due to vehicle’s movement, layering of DPM caused by the buoyancy effect, and backflow of diesel exhaust as a result of the low speed ventilation airflow. Miners in the vicinity should be aware of the high DPM regions and be protected accordingly as detailed below.

The general flow features showed the motion of the vehicle continuously altered this three-dimensional flow which in turn affected the DPM distribution. When driving upstream after loading, the truck driver will not be affected by the high DPM plume. The buoyancy effect, ventilation airflow and piston effect acting together flattened the diesel exhaust flow 25 m downstream from the truck engine and curved toward the roof. When driving downstream after loading, the exhaust flow from the truck cannot be clearly observed due to the high DPM fumes produced during loading. The truck drove into the high DPM plume and the driver need additional protection from the DPM.

For each case during the hauling operation, the LHD was assumed to be operating. The results showed that the high DPM plume occupied the entire face area and the intersection area. The LHD operator and any miners working in these regions need to wear personal protective equipment or work inside an environmental cab. Due to the buoyancy effect and low main airflow speed, a high DPM concentration plume can backflow toward the roof region of the upstream area, causing problems to miners working close to the roof if operating in the close upstream region.
